# Development and validation of an explainable machine learning model for predicting osteoporosis in patients with type 2 diabetes mellitus

**DOI:** 10.3389/fendo.2025.1611499

**Published:** 2025-08-07

**Authors:** Qipeng Wei, Zihao Liu, Xiaofeng Chen, Hao Li, Weijun Guo, Qingyan Huang, Jinxiang Zhan, Shiji Chen, Dongling Cai

**Affiliations:** ^1^ Department of Orthopedics, Panyu Hospital of Chinese Medicine, Guangzhou, China; ^2^ Panyu Hospital of Chinese Medicine, Guangzhou University of Chinese Medicine, Guangzhou, China

**Keywords:** osteoporosis, type 2 diabetes mellitus, explainable machine learning, predictive model, risk assessment

## Abstract

**Objective:**

Osteoporosis is a common complication in patients with type 2 diabetes mellitus (T2DM), yet its screening rate remains low. This study aimed to develop and validate a cost-effective and interpretable machine learning (ML) model to predict the risk of osteoporosis in patients with T2DM.

**Methods:**

This retrospective study included 1560 inpatients who underwent dual-energy X-ray absorptiometry (DXA) between January 2022 and December 2023 at Panyu Hospital of Chinese Medicine. Demographic information and laboratory test results obtained within 24 hours of hospital admission were collected. Potential predictive features were identified using univariate analysis, least absolute shrinkage and selection operator (LASSO) regression, and the Boruta algorithm. Eight supervised ML algorithms were applied to construct predictive models. Model performance was evaluated based on the area under the receiver operating characteristic curve (AUC), calibration plots, decision curve analysis (DCA), accuracy, sensitivity, specificity, and F1 score. The SHapley Additive exPlanations (SHAP) method was used to interpret the model and visualize feature importance.

**Results:**

Ten predictive features were selected based on the intersection of the three feature selection methods. Among the tested models, logistic regression achieved the best overall performance, with an AUC of 0.812, an accuracy of 0.762, a sensitivity of 0.809, a specificity of 0.761, and an F1 score of 0.771 in the validation set. Calibration plots and DCA curves demonstrated good agreement and the highest net clinical benefit. SHAP analysis identified age, sex, alkaline phosphatase, uric acid, hemoglobin, and neutrophil count as the six most influential features. An easy-to-use, web-based risk calculator was developed based on the logistic model and is available at: https://t2dm.shinyapps.io/t2dm-osteoporosis/.

**Conclusion:**

We developed an interpretable and accessible ML-based online tool that enables preliminary screening of osteoporosis risk in patients with T2DM using routine blood indicators. This tool may assist clinicians in early risk identification and reduce the underdiagnosis of osteoporosis.

## Introduction

Diabetes mellitus has emerged as a major global public health concern with significant impacts on morbidity and mortality. According to recent estimates, approximately 537 million people are currently living with diabetes worldwide. In China, the prevalence has reached 12.8%, affecting around 140 million individuals, with type 2 diabetes mellitus (T2DM) accounting for 90–95% of all cases (1).

Osteoporosis (OP), a chronic skeletal disorder characterized by trabecular deterioration, disrupted bone microarchitecture, decreased bone mass per unit volume, and increased bone fragility and fracture risk, represents one of the most common complications of diabetes (2). In patients with diabetes, hormonal imbalances and metabolic disturbances contribute to a range of complications. Persistent hyperglycemia accelerates calcium loss, disrupts bone metabolism, and leads to diabetic osteoporosis. Diabetic osteoporosis substantially increases the risk of falls and fractures, which in turn results in reduced quality of life and heightened mortality. In China, the prevalence of osteoporosis among individuals with T2DM is estimated at 37.8%, highlighting a critical yet frequently underrecognized public health issue (3). Accurate assessment of osteoporosis risk in patients with T2DM is therefore essential. However, a standardized and widely accepted risk assessment tool is currently lacking.

In recent years, machine learning (ML) has emerged as a powerful tool in medical diagnostics due to its ability to handle complex, high-dimensional data and uncover non-linear relationships between predictors and outcomes (4). In the field of metabolic bone disease, ML has shown promise in enhancing osteoporosis risk stratification by integrating diverse clinical and biochemical variables (5). A systematic review by Sadat-ali evaluated the performance of AI models in predicting osteoporotic fractures and illustrated that AI is a promising tool and that it may outperform conventional detection methods (6).However, many existing models require imaging inputs or are limited by interpretability. Therefore, applying interpretable ML approaches to routine clinical data offers a cost-effective and scalable solution to identify high-risk individuals, particularly in populations such as patients with T2DM, who are often under-screened.

In this study, we developed and validated an ML-based model to predict the risk of osteoporosis in patients with T2DM. The most effective predictive algorithm was identified through model comparison, and a user-friendly web-based tool was constructed to facilitate clinical application and personalized risk assessment.

## Methods

### Study design

**Figure 1:**
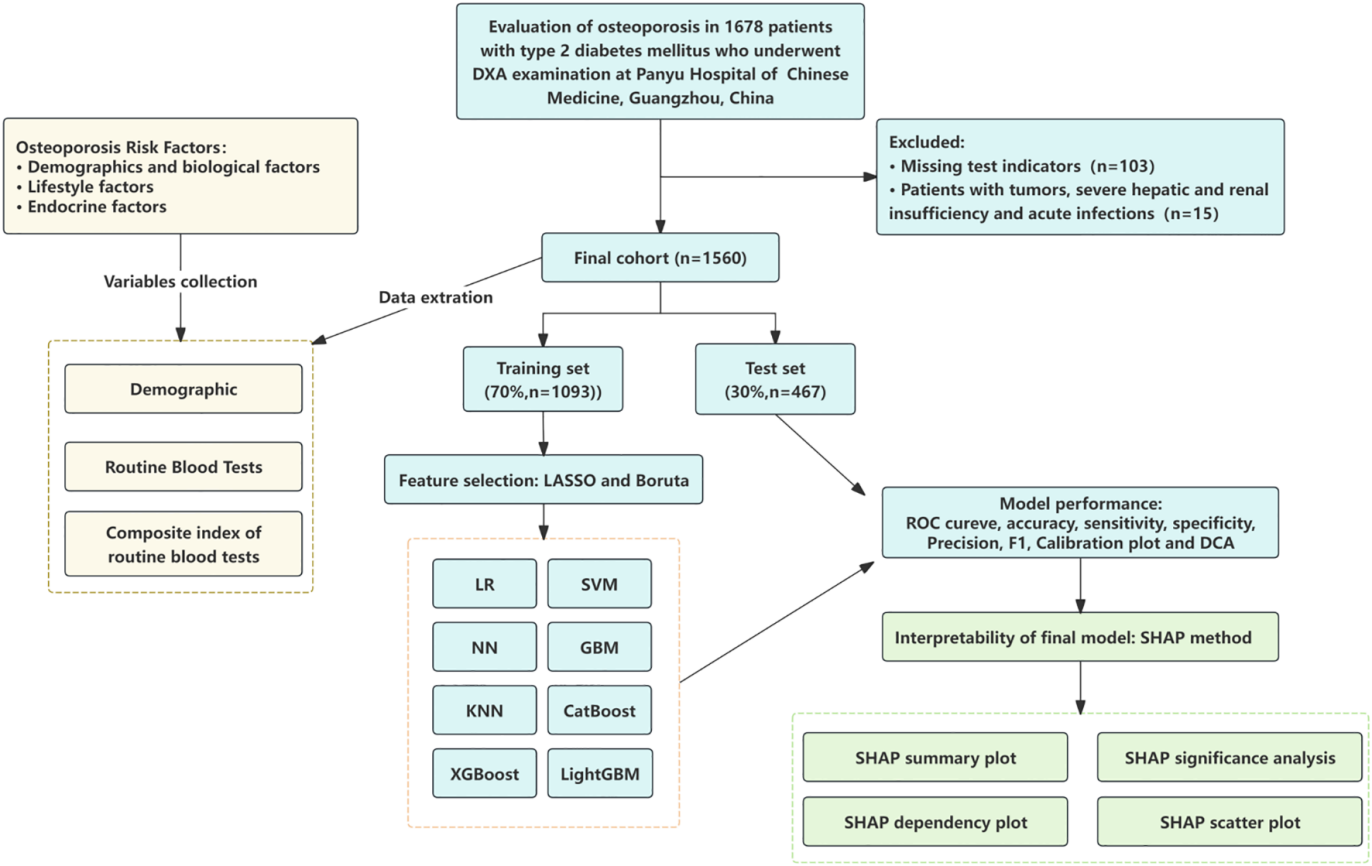
Study flow chart. LR, logistic regression; SVM, support vector machine; GBM, gradient boosting machine; NN, neural network; XGBoost, extreme gradient boosting; KNN, k-nearest neighbors; LightGBM, light gradient boosting machine; AdaBoost, adaptive boosting; ROC, receiver operating characteristic curves; DCA, decision curve analysis; SHAP, Shapley additive explanations.

This single-center, retrospective study consecutively enrolled hospitalized patients with type 2 diabetes mellitus (T2DM) who underwent dual-energy X-ray absorptiometry (DXA) at Panyu Hospital of Chinese Medicine between January 2022 and December 2023. Patients who underwent DXA examination during hospitalization were eligible for inclusion. The inclusion criteria were as follows: (1) age ≥ 45 years; (2) completed standardized DXA assessment; and (3) availability of complete electronic medical records, including demographic information and routine laboratory test results. Exclusion criteria included: (1) secondary osteoporosis; (2) hematological disorders; (3) history of malignancy; (4) severe hepatic or renal insufficiency; (5) acute infectious disease; and (6) incomplete clinical data.Bone mineral density (BMD) was measured using a standardized DXA protocol, assessing the lumbar spine (L1–L4), left femoral neck, and total hip. According to the diagnostic criteria defined by the World Health Organization, participants were classified into the osteoporosis group (T-score ≤ –2.5 SD) and the non-osteoporosis group (T-score > –2.5 SD) (**Figure 1**).

The study protocol adhered to the Declaration of Helsinki and was approved by the Institutional Review Board of Panyu Hospital of Guangzhou University of Chinese Medicine. Given the retrospective nature of this study, informed consent was waived.

### Data collection and preprocessing

Baseline demographic characteristics (age and sex) and standardized laboratory parameters were collected. Venous blood samples were collected from all fasting participants within 24 hours of hospital admission. Complete blood counts, including hemoglobin (HGB), neutrophil count (NEUT), red blood cell count (RBC), platelet count (PLT), lymphocyte count (LYMPH), and monocyte count (MONO), were analyzed using the Mindray BC-6800Plus hematology analyzer. Biochemical analyses included total cholesterol (TC), triglycerides (TG), high-density lipoprotein cholesterol (HDL-C), low-density lipoprotein cholesterol (LDL-C), alkaline phosphatase (ALP), alanine aminotransferase (ALT), albumin (ALB), uric acid (UA), fasting blood glucose (FBG), serum calcium (Ca), serum phosphate (Pi), and creatinine (Cr). All laboratory evaluations and DXA measurements were performed during the same hospitalization period.

Before model development, several preprocessing steps were performed to ensure data quality and consistency. Duplicate records and entries with apparent input errors were excluded. For variables with missing values less than 20%, imputation was performed using the mean for continuous variables and the mode for categorical variables. Variables with more than 20% missing data were excluded from model construction.

### Calculation of derived biomarkers

To capture complex interactions related to metabolic and inflammatory status, we derived seven composite indices from the original laboratory values: Monocyte-to-HDL ratio (MHR), Neutrophil-to-HDL ratio (NHR), Platelet-to-HDL ratio (PHR), Lymphocyte-to-HDL ratio (LHR), Triglyceride-Glucose index (TyG), Cholesterol-Glucose index (CHG), and Non-HDL-to-Neutrophil ratio (NHHR). These indices have been previously associated with chronic inflammation, insulin resistance, and cardiovascular risk—factors that are also implicated in osteoporosis, particularly among patients with type 2 diabetes mellitus.Seven novel indices were derived from original laboratory measurements using the following formulas, as previously described in the literature (7–13):


$$\text{Monocyte}-\text{to}-\text{HDL Ratio }\left(\text{MHR}\right):\text{ MONO }\left(\times 10^{9}/\text{L}\right)/\text{HDL}-\text{C }\left(\text{mmol}/\text{L}\right)$$

$$\text{Neutrophil}-\text{to}-\text{HDL Ratio }\left(\text{NHR}\right):\text{ NEUT }\left(\times 10^{9}/\text{L}\right)/\text{HDL}-\text{C }\left(\text{mmol}/\text{L}\right)$$

$$\text{Platelet}-\text{to}-\text{HDL Ratio }\left(\text{PHR}\right):\text{ PLT }\left(\times 10^{9}/\text{L}\right)/\text{HDL}-\text{C }\left(\text{mmol}/\text{L}\right)$$

$$\text{Lymphocyte}-\text{to}-\text{HDL Ratio }\left(\text{LHR}\right):\text{ LYMPH }\left(\times 10^{9}/\text{L}\right)/\text{HDL}-\text{C }\left(\text{mmol}/\text{L}\right)$$

Triglyceride−Glucose Index (TyG): ln[TG (mg/dL) × FPG (mg/dL)/2]

$$\text{Non}-\text{HDL}-\text{to}-\text{Neutrophil Ratio }\left(\text{NHHR}\right):\;\left[\text{TC }\left(\text{mg}/\text{dL}\right)\;-\text{ HDL}-\text{C }\left(\text{mg}/\text{dL}\right)\right]/\text{HDL}-\text{C }\left(\times 10^{9}/\text{L}\right)$$

Cholesterol−Glucose Index (CHG): ln[TC (mg/dL) × FPG (mg/dL)/(2 × HDL−C (mg/dL))]

### Feature selection and model construction

In this study, the caret package in R was used to randomly partition the dataset into a training set (70%) and a testing set (30%). The training set was utilized for model development, while the testing set was reserved for performance evaluation.

To identify potential predictors from baseline variables in the training set, three independent feature selection methods were employed: univariate analysis, least absolute shrinkage and selection operator (LASSO) regression, and the Boruta algorithm (14). Univariate analysis, a conventional statistical method, selected variables with a P-value < 0.05. LASSO regression identified predictors with non-zero coefficients, effectively addressing multicollinearity and reducing the risk of model overfitting due to excessive inter-variable correlation (15). In this study, LASSO regression with 10-fold cross-validation was performed to screen variables from high-dimensional data.The Boruta algorithm, a wrapper method based on feature importance, identifies relevant variables by comparing the Z-scores of actual features to those of permuted “shadow” features. In each iteration, Z-scores for the real features were computed using a random forest (RF) classifier, while those for the shadow features were obtained by randomly shuffling the original variables (16). Features consistently performing worse than the shadow features were iteratively removed.To ensure that there was no multicollinearity among the candidate variables, we calculated the variance inflation factor (VIF) scores. Variables with high collinearity (VIF > 10) were excluded to maintain model stability.

The intersection of variables selected by all three methods was used to develop prediction models. Ten supervised machine learning algorithms were implemented for model construction: logistic regression (LR), support vector machine (SVM), gradient boosting machine (GBM), neural network (NN), extreme gradient boosting (XGBoost), k-nearest neighbors (KNN), AdaBoost, and LightGBM.

### Model evaluation

The optimal model was determined through a comprehensive evaluation of the discriminative ability, calibration performance, and clinical applicability of all ten candidate models. The receiver operating characteristic (ROC) curve was constructed to visually represent the model’s discriminative power, with the area under the curve (AUC) serving as the primary quantitative metric. Additional performance indicators, including accuracy, sensitivity, specificity, precision, and F1 score, were also calculated to supplement the evaluation. Calibration and clinical utility were further assessed using decision curve analysis (DCA) and calibration plots, respectively.

### Model interpretation

To enhance model interpretability, Shapley Additive Explanations (SHAP) values were analyzed to quantify the contribution and importance of each feature in determining the final classification outcome. A higher SHAP value indicates greater influence on the model’s output prediction. We present a feature importance analysis based on SHAP values to interpret the results of the optimal model (17).

### Statistical analysis

All statistical analyses were conducted using R software (version 4.4.3). Continuous variables with a normal distribution were presented as mean ± standard deviation (SD), while non-normally distributed continuous variables were expressed as median with interquartile range (IQR). Categorical variables were summarized as frequencies and percentages. Group differences in continuous variables were assessed using either the independent samples t-test or the Kruskal–Wallis test, as appropriate. Categorical variables were compared using the chi-square test or Fisher’s exact test. A two-sided p-value < 0.05 was considered statistically significant.

Lasso regression and Boruta feature selection were performed using the “glmnet” and “Boruta” R packages, respectively. Predictive model development and training were conducted with the “caret” package, with default hyperparameter tuning implemented via grid search. SHAP value analysis for model interpretation was carried out using the “shapviz” package.

## Results

### Comparison of clinical characteristics

A total of 1,560 patients were included in this study and randomly assigned to the training set (n = 1,093) and the testing set (n = 467) in a 7:3 ratio. No statistically significant differences were observed between the two sets across all clinical variables (P > 0.05), as detailed in **Table 1**.

**Table 1:** Baseline characteristics of patients.

Variables	Total (n = 1560)	Training set (n = 1093)	Test set (n = 467)	*P*
Gender(%)				0.584
Female	1147 (73.53)	808 (73.92)	339 (72.59)	
Male	413 (26.47)	285 (26.08)	128 (27.41)	
Age	70.00 (63.00, 77.00)	69.00 (63.00, 77.00)	70.00 (63.00, 77.00)	0.477
NEUT(×10^9^/L)	4.72 (3.57, 6.32)	4.68 (3.60, 6.39)	4.78 (3.42, 6.21)	0.564
HGB(×10^9^/L)	126.00 (115.00, 137.00)	127.00 (115.00, 137.00)	125.00 (115.00, 137.00)	0.546
LYMPH(×10^9^/L)	1.72 (1.28, 2.21)	1.72 (1.28, 2.21)	1.73 (1.29, 2.21)	0.575
PLT,(×10^9^/L)	232.00 (191.00, 276.00)	233.00 (188.00, 276.00)	229.00 (195.50, 276.00)	0.869
MONO(×10^9^/L)	0.47 (0.37, 0.58)	0.46 (0.37, 0.58)	0.47 (0.38, 0.58)	0.746
RBC(×10^9^/L)	4.32 (3.93, 4.70)	4.32 (3.94, 4.72)	4.29 (3.90, 4.69)	0.431
TC(mmol/L)	4.49 (3.76, 5.36)	4.49 (3.76, 5.35)	4.47 (3.75, 5.42)	0.921
TG(mmol/L)	1.46 (1.03, 2.07)	1.43 (1.02, 2.03)	1.55 (1.05, 2.09)	0.215
HDL_C(mmol/L)	1.17 (0.99, 1.41)	1.17 (0.99, 1.41)	1.17 (0.99, 1.40)	0.798
LDL_C(mmol/L)	2.74 (2.08, 3.47)	2.74 (2.10, 3.47)	2.74 (2.04, 3.50)	0.869
ALP(U/L)	74.00 (61.00, 90.00)	73.00 (61.00, 90.00)	75.00 (61.00, 91.90)	0.540
ALT(U/L)	16.60 (12.00, 24.00)	16.30 (12.00, 24.00)	17.00 (12.00, 23.15)	0.701
AST(U/L)	18.00 (15.00, 22.00)	18.00 (15.00, 22.00)	18.00 (14.10, 22.00)	0.909
ALB(g/L)	39.40 (36.60, 41.90)	39.30 (36.50, 42.00)	39.60 (36.90, 41.80)	0.549
Cr(μmol/L)	66.00 (54.00, 84.00)	66.00 (54.00, 85.00)	67.00 (55.00, 82.00)	0.736
UA(μmol/L)	324.50 (266.00, 408.00)	323.00 (265.00, 406.00)	327.00 (267.50, 409.50)	0.356
Ca(mmol/L)	2.28 (2.21, 2.35)	2.28 (2.20, 2.35)	2.28 (2.21, 2.35)	0.404
Pi(mmol/L)	1.15 (1.03, 1.28)	1.15 (1.03, 1.27)	1.16 (1.03, 1.29)	0.624
FBG(mmol/L)	7.19 (5.80, 9.45)	7.26 (5.83, 9.54)	6.99 (5.71, 9.36)	0.251
MHR	0.40 (0.30, 0.53)	0.40 (0.30, 0.53)	0.40 (0.31, 0.53)	0.665
NHR	4.07 (2.92, 5.71)	4.08 (2.91, 5.79)	4.06 (2.94, 5.50)	0.646
PHR	195.88 (151.61, 249.09)	196.30 (150.76, 249.55)	195.19 (154.92, 248.78)	0.602
LHR	1.46 (1.03, 1.99)	1.46 (1.01, 1.98)	1.46 (1.06, 2.02)	0.451
TYG	9.05 (8.63, 9.54)	9.05 (8.64, 9.54)	9.06 (8.61, 9.54)	0.894
NHHR	2.78 (2.03, 3.68)	2.78 (2.03, 3.68)	2.80 (2.00, 3.69)	0.881
CHG	5.52 (5.19, 5.90)	5.53 (5.20, 5.90)	5.51 (5.17, 5.90)	0.561

Data are shown as median with interquartile range (IQR) for continuous variables and number with percentage for categorical variables

HGB, hemoglobin; NEUT, neutrophil count; RBC, red blood cell count; PLT, platelet count; LYMPH, lymphocyte count; MONO, monocyte count; TC, total cholesterol; TG, triglycerides; HDL-C, high-density lipoprotein cholesterol; LDL-C, low-density lipoprotein cholesterol; ALP, alkaline phosphatase; ALT, alanine aminotransferase; ALB, albumin; UA, uric acid; FBG, fasting plasma glucose; Ca, calcium; Pi, phosphorus; Cr, creatinine; MHR, Monocyte-to-HDL Ratio; NHR, Neutrophil-to-HDL Ratio; PHR, Platelet-to-HDL Ratio; LHR, Lymphocyte-to-HDL Ratio; TyG, Triglyceride-Glucose Index; NHHR, Non-HDL-to-Neutrophil Ratio; CHG, Cholesterol-Glucose Index.

In the training cohort, patients with and without osteoporosis showed statistically significant differences in several clinical and biochemical variables, as detailed in **Table 2**. These included age, sex, hemoglobin (HGB), neutrophil count (NEUT), lymphocyte count (LYMPH), red blood cell count (RBC), triglycerides (TG), high-density lipoprotein cholesterol (HDL-C), alkaline phosphatase (ALP), alanine aminotransferase (ALT), albumin (ALB), uric acid (UA), serum calcium (Ca), as well as derived indices such as neutrophil-to-HDL ratio (NHR), platelet-to-HDL ratio (PHR), lymphocyte-to-HDL ratio (LHR), triglyceride-glucose index (TyG), and non-HDL-to-neutrophil ratio (NHHR) (all P < 0.05). Detailed comparisons are provided in **Table 2**. Conversely, no statistically significant differences were found in platelet count (PLT), monocyte count (MONO), total cholesterol (TC), low-density lipoprotein cholesterol (LDL-C), aspartate aminotransferase (AST), creatinine (Cr), serum phosphate (Pi), fasting blood glucose (FBG), monocyte-to-HDL ratio (MHR), and cholesterol-glucose index (CHG) between the two groups (all P > 0.05).

**Table 2:** Results of univariate analysis.

Variables	Total (n = 1093)	Osteoporosis (n = 595)	Non-Osteoporosis (n = 498)	*P*
Gender(%)				<.001
Female	808 (73.92)	508 (85.38)	300 (60.24)	
Male	285 (26.08)	87 (14.62)	198 (39.76)	
Age	69.00 (63.00, 77.00)	73.00 (66.00, 79.00)	66.00 (60.00, 72.00)	<.001
NEUT(×10^9^/L)	4.68 (3.60, 6.39)	5.09 (3.73, 7.16)	4.34 (3.51, 5.80)	<.001
HGB(×10^9^/L)	127.00 (115.00, 137.00)	123.00 (110.00, 132.50)	131.00 (120.25, 141.00)	<.001
LYMPH(×10^9^/L)	1.72 (1.28, 2.21)	1.64 (1.17, 2.11)	1.82 (1.36, 2.33)	<.001
PLT,(×10^9^/L)	233.00 (188.00, 276.00)	233.00 (188.00, 279.00)	233.00 (189.25, 272.75)	0.785
MONO(×10^9^/L)	0.46 (0.37, 0.58)	0.47 (0.37, 0.59)	0.46 (0.37, 0.57)	0.470
RBC(×10^9^/L)	4.32 (3.94, 4.72)	4.27 (3.85, 4.59)	4.44 (4.06, 4.81)	<.001
TC(mmol/L)	4.49 (3.76, 5.35)	4.48 (3.83, 5.29)	4.50 (3.69, 5.44)	0.983
TG(mmol/L)	1.43 (1.02, 2.03)	1.32 (0.98, 1.81)	1.59 (1.12, 2.28)	<.001
HDL_C(mmol/L)	1.17 (0.99, 1.41)	1.20 (1.02, 1.42)	1.12 (0.96, 1.39)	<.001
LDL_C(mmol/L)	2.74 (2.10, 3.47)	2.75 (2.17, 3.42)	2.72 (2.06, 3.51)	0.648
ALP(U/L)	73.00 (61.00, 90.00)	76.00 (64.00, 95.05)	69.05 (57.00, 83.22)	<.001
ALT(U/L)	16.30 (12.00, 24.00)	15.00 (11.00, 22.00)	18.00 (13.00, 25.00)	<.001
AST(U/L)	18.00 (15.00, 22.00)	18.00 (14.85, 21.95)	18.00 (15.00, 22.48)	0.263
ALB(g/L)	39.30 (36.50, 42.00)	38.90 (35.70, 41.60)	40.00 (37.40, 42.30)	<.001
Cr(μmol/L)	66.00 (54.00, 85.00)	64.00 (53.00, 84.00)	68.00 (55.00, 85.75)	0.089
UA(μmol/L)	323.00 (265.00, 406.00)	311.00 (253.50, 386.00)	338.50 (286.00, 420.00)	<.001
Ca(mmol/L)	2.28 (2.20, 2.35)	2.27 (2.19, 2.34)	2.29 (2.22, 2.36)	<.001
Pi(mmol/L)	1.15 (1.03, 1.27)	1.14 (1.02, 1.27)	1.16 (1.04, 1.29)	0.089
FBG(mmol/L)	7.26 (5.83, 9.54)	7.40 (5.96, 9.64)	7.12 (5.69, 9.26)	0.057
MHR	0.40 (0.30, 0.53)	0.39 (0.29, 0.53)	0.40 (0.31, 0.53)	0.157
NHR	4.08 (2.91, 5.79)	4.26 (2.98, 6.12)	3.92 (2.84, 5.44)	0.010
PHR	196.30 (150.76, 249.55)	190.77 (143.56, 241.98)	201.03 (157.50, 255.48)	0.016
LHR	1.46 (1.01, 1.98)	1.35 (0.92, 1.85)	1.57 (1.19, 2.15)	<.001
TYG	9.05 (8.64, 9.54)	8.99 (8.59, 9.49)	9.12 (8.70, 9.60)	0.002
NHHR	2.78 (2.03, 3.68)	2.69 (2.00, 3.47)	2.88 (2.07, 3.85)	0.003
CHG	5.53 (5.20, 5.90)	5.52 (5.20, 5.87)	5.55 (5.20, 5.93)	0.609

Data are shown as median with interquartile range (IQR) for continuous variables and number with percentage for categorical variables.

HGB, hemoglobin; NEUT, neutrophil count; RBC, red blood cell count; PLT, platelet count; LYMPH, lymphocyte count; MONO, monocyte count; TC, total cholesterol; TG, triglycerides; HDL-C, high-density lipoprotein cholesterol; LDL-C, low-density lipoprotein cholesterol; ALP, alkaline phosphatase; ALT, alanine aminotransferase; ALB, albumin; UA, uric acid; FBG, fasting plasma glucose; Ca, calcium; Pi, phosphorus; Cr, creatinine; MHR, Monocyte-to-HDL Ratio; NHR, Neutrophil-to-HDL Ratio; PHR, Platelet-to-HDL Ratio; LHR, Lymphocyte-to-HDL Ratio; TyG, Triglyceride-Glucose Index; NHHR, Non-HDL-to-Neutrophil Ratio; CHG, Cholesterol-Glucose Index.

### Model development

**Figure 2:**
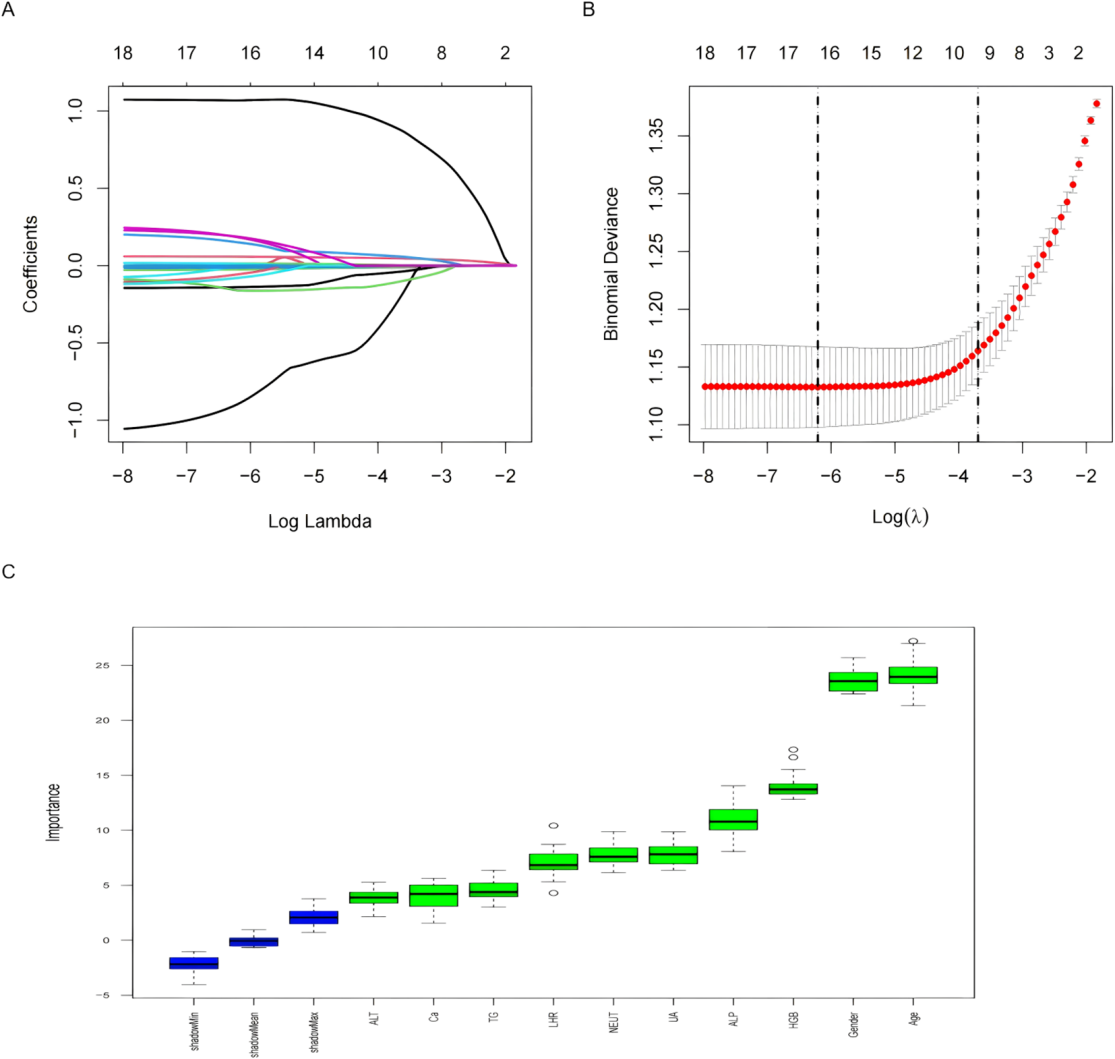
Features selected by univariate analysis, Lasso and Boruta. **(A)** The Lasso regression coefficient profiles of characteristics. **(B)** The optimal lambda selection in the Lasso regression with 10-fold cross-validation. **(C)** Variables selected by Boruta algorithm.

Eighteen variables identified through univariate analysis were subjected to further feature selection using LASSO regression and the Boruta algorithm. In the LASSO regression, the optimal lambda value was determined to be 0.024, which yielded 10 key predictive features. These included two demographic variables (age and sex) and eight laboratory indicators (ALP, UA, HGB, NEUT, ALT, LHR, Ca, and TG). Notably, the results of the Boruta algorithm fully corroborated those of LASSO regression, confirming the same 10 features (**Figure 2**). The candidate variables also showed VIF values below the accepted threshold of 10, indicating no significant Multicollinearity.These variables were ultimately selected as the core predictors for model construction.

### Model evaluation

Eight machine learning algorithms were employed to develop models for predicting osteoporosis in patients with T2DM: logistic regression (LR), support vector machine (SVM), gradient boosting machine (GBM), neural network (NN), extreme gradient boosting (XGBoost), k-nearest neighbors (KNN), AdaBoost, and LightGBM. Among these, the LR model demonstrated the most robust and consistent performance in the testing set (**Table 3**).

**Table 3:** The prediction performance of each model.

Model	AUC (95% CI)	Accuracy	Sensitivity	Specificity	Precision	F1
Training set						
Logistic	0.835 (0.780~0.840)	0.791	0.812	0.791	0.804	0.795
SVM	0.767 (0.740~0.795)	0.709	0.746	0.665	0.727	0.736
GBM	0.873 (0.852~0.893)	0.804	0.805	0.803	0.83	0.817
NeuralNetwork	0.797 (0.771~0.823)	0.724	0.736	0.709	0.751	0.744
Xgboost	0.809 (0.784~0.835)	0.739	0.746	0.731	0.768	0.757
KNN	0.932 (0.918~0.945)	0.847	0.845	0.849	0.87	0.858
Adaboost	0.711 (0.683~0.739)	0.637	0.461	0.847	0.783	0.58
LightGBM	0.912 (0.895~0.929)	0.844	0.866	0.819	0.851	0.858
Test set						
Logistic	0.812 (0.750~0.832)	0.762	0.809	0.761	0.778	0.771
SVM	0.773 (0.731~0.815)	0.722	0.65	0.808	0.801	0.717
GBM	0.780 (0.739~0.822)	0.713	0.677	0.756	0.768	0.72
NeuralNetwork	0.777 (0.735~0.819)	0.715	0.697	0.737	0.76	0.727
Xgboost	0.771 (0.728~0.814)	0.728	0.685	0.779	0.787	0.733
KNN	0.734 (0.689~0.779)	0.685	0.717	0.648	0.708	0.712
Adaboost	0.717 (0.674~0.761)	0.704	0.78	0.615	0.707	0.606
LightGBM	0.723 (0.676~0.770)					0.742

**Figure 3:**
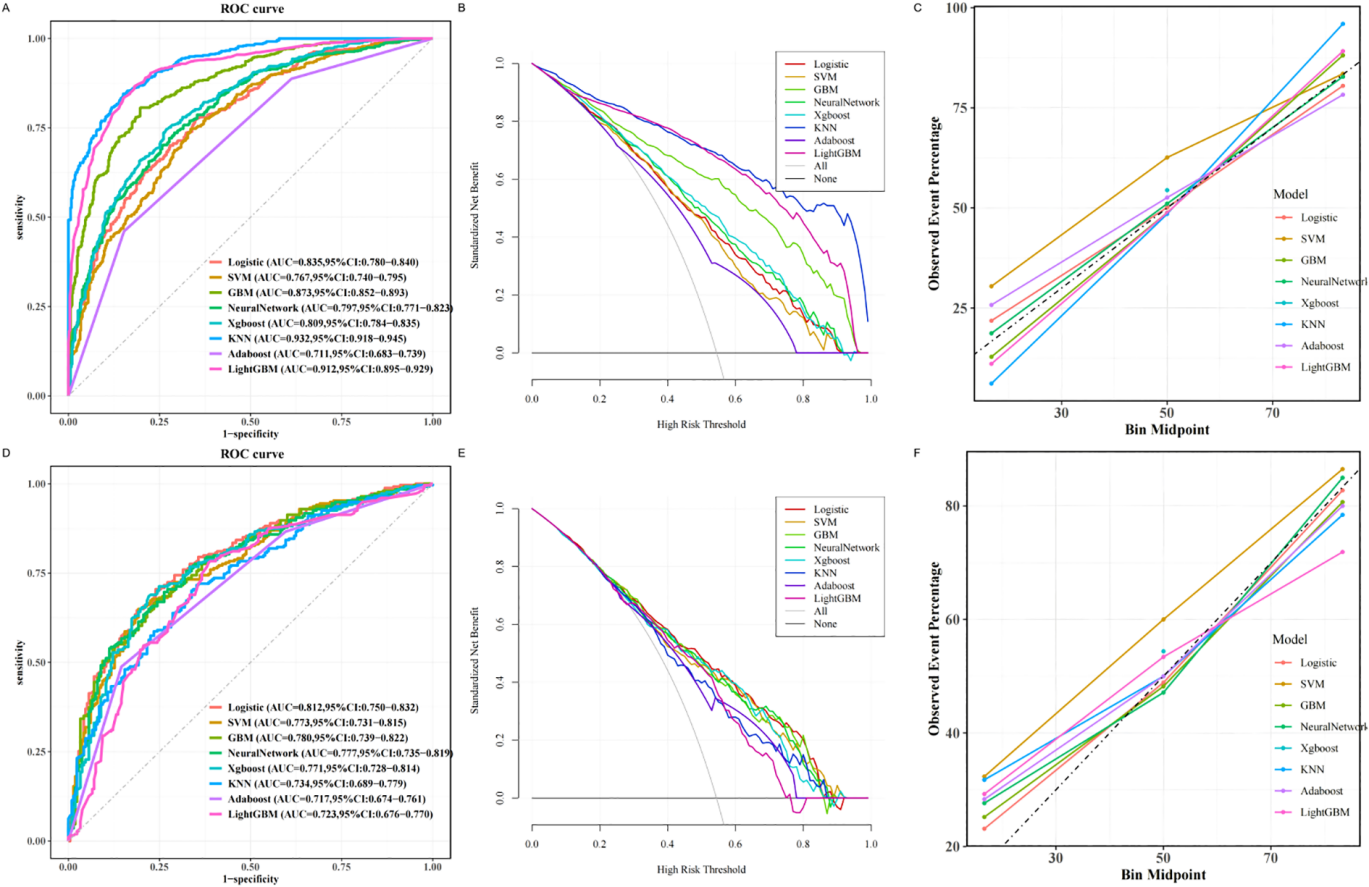
Machine learning based prediction model for osteoporosis. **(A)** ROC curve of the training set of the machine learning-based model. **(B)** Training set DCA of the machine learning-based model. **(C)** Training set calibration curve of the machine learning based model. **(D)** Testing set ROC curve of machine learning based model. **(E)** Testing set DCA based on machine learning model. **(F)** Testing set calibration curve based on machine learning model. ROC, receiver operating characteristic; DCA, decision curve analysis.

As illustrated in **Figure 3**, the LR model achieved the highest discriminative performance, with an AUC of 0.812 (95% CI: 0.750–0.832). It also outperformed other models in terms of accuracy (0.762), sensitivity (0.809), and F1 score (0.771), while maintaining favorable specificity (0.761) and precision (0.778). In comparison, KNN (AUC = 0.734, 95% CI: 0.689–0.779), AdaBoost (AUC = 0.717, 95% CI: 0.674–0.761), and LightGBM (AUC = 0.723, 95% CI: 0.676–0.770) demonstrated inferior classification performance. Although the SVM model achieved slightly higher specificity (0.808) and precision (0.801), its lower sensitivity (0.650), F1 score (0.717), and AUC (0.773, 95% CI: 0.731–0.815) indicated limited generalizability. Moreover, the GBM, NN, and XGBoost models exhibited noticeably lower AUCs, accuracy, sensitivity, and F1 scores compared to the LR model, further supporting the superior generalization ability of the latter.

### Model interpretability and web application

**Figure 4:**
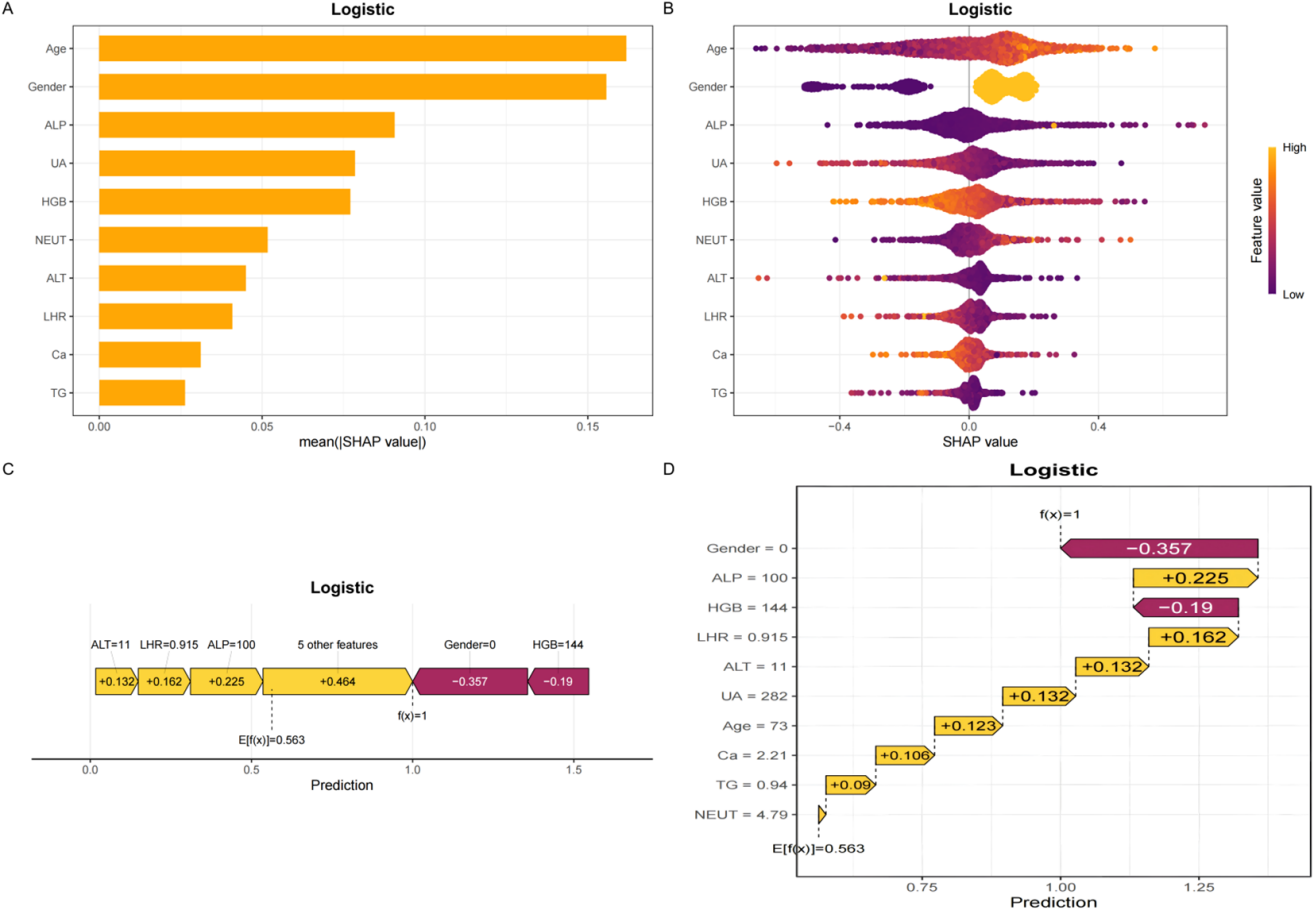
SHAP plots. **(A)** Bar chart of the mean absolute SHAP value for each predictor of the Logistic model in descending order. **(B)** SHAP summary plot shows feature importance for each predictor of the Logistic model in descending order. The upper predictors are more important to the model’s predictive outcome. A dot is created for each feature attribution value for the Logistic model of each patient. The further away a dot is from the baseline SHAP value of zero, the stronger it effects the model output. Dots are colored according to the values of features. Yellow represents higher feature values and red represents lower feature values. **(C, D)** The force plots provide personalized feature attributions using representative examples. SHAP, Shapley additive explanations.

**Figure 5:**
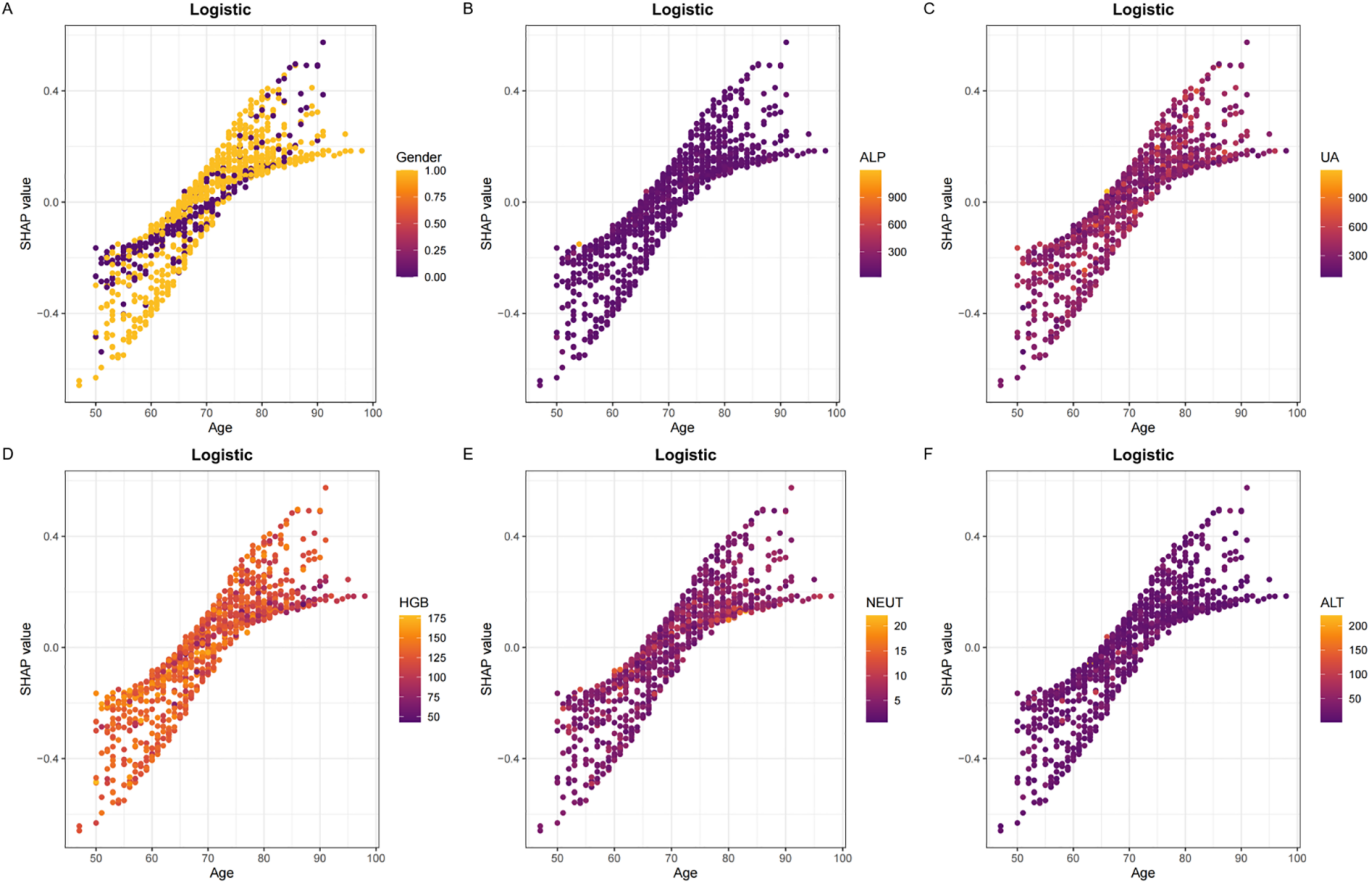
Panels **(A–F)** display the SHAP dependency plots for features in the Logistic model, illustrating their relationships with Age.The Y-axis represents SHAP values, while the X-axis represents actual clinical parameters. Significantly, when a feature's SHAP value is greater than 0, it suggests an increased risk of osteoporosis, whereas a negative SHAP value suggests a reduced risk.

To elucidate the decision-making process of the LR model, we applied the SHapley Additive exPlanations (SHAP) framework for both global and local interpretation. The bar chart in **Figure 4A** displays the mean absolute SHAP values for each predictor, reflecting their overall contribution to the model.The SHAP summary plot (**Figure 4B**) ranked the importance of predictive features, with age, sex, ALP, UA, HGB, NEUT, ALT, LHR, Ca, and TG identified as the most influential variables in descending order. **Figures 4C, D** provide further insights into how individual SHAP values influence predictions. Age emerged as the most dominant predictor, and interaction plots (**Figures 5A–F**) illustrated the complex interrelationships between age and other variables.

To enhance the model’s clinical utility and streamline its application in practice, we optimized the input requirements by limiting them to the 10 key features, many of which are routinely obtained in standard blood tests. Furthermore, we developed a user-friendly, web-based calculator (available at:https://t2dm.shinyapps.io/t2dm-osteoporosis/) to facilitate real-time risk assessment of osteoporosis in patients with T2DM. This platform enables clinicians to enter patient data and receive immediate, individualized risk estimations, thereby supporting early intervention and personalized care strategies.

## Discussion

In this study, we developed and validated a machine learning model for predicting osteoporosis in patients with type 2 diabetes mellitus (T2DM), using demographic characteristics and routine blood test indicators. According to the latest IDF Diabetes Atlas, the global prevalence of diabetes has reached approximately 643 million people, meaning that one in nine adults is affected (18). In China alone, between 50% and 66% of adults with T2DM exhibit decreased bone mineral density (BMD), and nearly one-third can be diagnosed with osteoporosis (19, 20). Therefore, there is an urgent need to establish a reliable screening tool specifically targeting osteoporosis in T2DM patients.

The logistic regression model constructed in this study demonstrated strong and consistent predictive performance in both the training and testing cohorts, with area under the curve (AUC) values ranging from 0.812 to 0.835. The high AUC values highlight the model’s accuracy and robustness in predicting osteoporosis among T2DM patients. Moreover, calibration curves and decision curve analysis (DCA) further confirmed the model’s good calibration and substantial clinical net benefit.

To enhance the model’s interpretability and clinical applicability, we employed a multistep feature selection process. Initially, univariate analysis was used to identify potential predictors. LASSO regression was then applied to address multicollinearity and eliminate irrelevant variables. Finally, the Boruta algorithm, based on random forest importance scores, was used to confirm the stability of selected features (15, 21, 22). This process identified 10 core predictors: age, sex, ALP, UA, HGB, NEUT, ALT, LHR, Ca, and TG. By reducing the number of variables, the logistic regression model became more interpretable and user-friendly, facilitating easier data collection in clinical settings.

Although more complex algorithms such as KNN and LightGBM demonstrated high discriminative ability in the training set (AUCs of 0.932 and 0.912, respectively), their substantial performance decline in the test set (AUCs of 0.734 and 0.723) indicated overfitting and limited generalizability. Overfitting, a common concern in machine learning models with small or moderate-sized datasets, compromises the reliability of predictions when applied to new data (23). In contrast, logistic regression maintained more stable performance across both sets (AUC from 0.835 to 0.812) and achieved a strong F1 score, indicating a balanced trade-off between precision and recall. Considering the goal of developing an interpretable and clinically applicable tool, we prioritized robustness and interpretability over algorithmic complexity. Logistic regression provides transparency in feature contributions and aligns better with clinical decision-making workflows, supporting its adoption as the final model.

The pathogenesis of osteoporosis in T2DM is multifactorial, involving aging, sex, metabolic dysregulation, chronic inflammation, and impaired bone remodeling (2, 24–27). Aging disrupts bone homeostasis by impairing osteoblast function, and the abrupt decline in estrogen levels after menopause accelerates osteoclast activation (28, 29). Hyperglycemia-induced oxidative stress increases skeletal fragility (30). Elevated ALP levels reflect increased bone turnover, potentially related to compensatory bone formation following bone microarchitecture disruption by advanced glycation end products (AGEs) (31). Reduced serum calcium may be attributed to insulin resistance and vitamin D deficiency, both of which disturb calcium-phosphorus metabolism and promote bone resorption (32, 33).

Inflammation also plays a critical role—elevated NEUT indicates systemic inflammation, which promotes osteoclast differentiation via pro-inflammatory cytokines such as IL-6 and TNF-α (34). Reduced HGB levels, indicative of anemia, may contribute to hypoxia and nutritional deficiencies, impairing bone repair capacity (35). UA plays a complex role in bone metabolism, particularly in patients with T2DM.While uric acid may exert antioxidant effects at physiological levels, elevated levels in patients with T2DM often indicate an adverse metabolic state that contributes to bone loss and increased fracture risk (36). From a metabolic perspective, while UA has antioxidant properties, its pro-inflammatory effects may counteract its protective role (37). Elevated ALT levels may impair vitamin D activation, thereby affecting calcium absorption (38). Both TG and LHR contribute to lipotoxicity, inhibiting osteoblast differentiation and promoting adipogenesis, ultimately accelerating bone loss (39).These mechanisms—driven by insulin resistance, oxidative stress, and AGEs accumulation—interact to form a complex “metabolism-inflammation-bone loss” network, leading to reduced bone density and increased fracture risk. SHAP-based interpretability analysis further validated the biological plausibility of our model’s predictions.

By integrating demographic and routine biochemical data, our logistic regression model achieved an AUC of 0.812 in the testing set—superior to previous models—and elucidated the intricate interplay among metabolic disturbances, chronic inflammation, and bone loss (40). Compared to the Osteoporosis Self-assessment Tool for Asians (OSTA), a widely used screening tool in Asian populations, our model demonstrated superior predictive performance. OSTA relies solely on age and body weight, and previous studies have reported an AUC of 0.736, with a sensitivity of 73.1% and specificity of 69.8% for identifying osteoporosis (T-score ≤ –2.5). However, OSTA does not incorporate metabolic or biochemical indicators, which are particularly relevant in patients with T2DM who often present with complex metabolic profiles. In contrast, our logistic regression model achieved an AUC of 0.812 and a sensitivity of 80.9%, outperforming OSTA by leveraging routinely available laboratory parameters (41).This model offers a practical approach for the early identification of osteoporosis in T2DM patients, enabling clinicians to initiate personalized management strategies. Although dual-energy X-ray absorptiometry (DXA) remains the gold standard for diagnosing osteoporosis, its availability is limited, especially in primary care settings (42). Our model can serve as an intelligent prescreening tool before DXA testing, facilitating early diagnosis and intervention.

To enhance clinical usability, we developed an online calculator based on the final logistic regression model. The tool requires input of only ten routine clinical variables,which are commonly available in patients with T2DM. This design allows for rapid, cost-effective screening without the need for imaging or additional lab tests. The model has been applied to the screening of osteoporosis in orthopedic inpatients in our hospital who may benefit from further DXA evaluation.

## Limitations

This study has several limitations. First, it was a single-center retrospective study, which may introduce selection bias and limit the generalizability of the findings. Differences in clinical practices, population characteristics, and laboratory standards across institutions may affect model performance. Therefore, external validation using multi-center and prospective cohorts is essential to assess the robustness and applicability of the model in broader clinical settings. Additionally, due to the retrospective design, some potentially relevant variables—such as height and weight—were not available and could not be included in the analysis, which may have influenced model accuracy.In the future, we intend to conduct a multicenter prospective study and seamlessly integrate the model into the hospital information system to achieve real-time risk stratification of osteoporosis in patients with type 2 diabetes. This will support early identification and timely referral for DXA assessment.

## Conclusion

In summary, we developed and validated an interpretable machine learning model based on routinely collected clinical and laboratory data to predict osteoporosis risk in patients with type 2 diabetes mellitus. The logistic regression model demonstrated favorable predictive performance, outperforming traditional screening tools, and provides a practical approach for early identification of patients at high risk for osteoporosis. By integrating commonly available biomarkers, the model facilitates cost-effective and accessible screening, especially in settings where DXA is unavailable or limited. Furthermore, the development of an online calculator enhances the model’s clinical utility by enabling easy risk assessment in real time.

## Data Availability

The original contributions presented in the study are included in the article/supplementary material. Further inquiries can be directed to the corresponding author.

## Glossary

ML: Machine Learning
T2DM: Type 2 Diabetes Mellitus
OP: Osteoporosis
DXA: Dual-energy X-ray Absorptiometry
BMD: Bone mineral density
DCA: Decision Curve Analysis
AUC: Area under the receiver operating characteristic curve
VIF: Variance inflation factor
SHAP: SHapley Additive exPlanations
HGB: Hemoglobin
NEUT: Neutrophil count
RBC: Red blood cell count
PLT: Platelet count
LYMPH: Lymphocyte count
MONO: Monocyte count
TC: Total cholesterol
TG: Triglycerides
HDL-C: High-density lipoprotein cholesterol
LDL-C: Low-density lipoprotein cholesterol
ALP: Alkaline phosphatase
ALT: Alanine aminotransferase
ALB: Albumin
UA: Uric acid
FBG: Fasting blood glucose
Ca: Serum calcium
Cr: Creatinine
Pi: Serum phosphate
MHR: Monocyte-to-HDL ratio
NHR: Neutrophil-to-HDL ratio
PHR: Platelet-to-HDL ratio
LHR: Lymphocyte-to-HDL ratio
TyG: Triglyceride-Glucose index
CHG: Cholesterol-Glucose index
NHHR: Non-HDL-to-Neutrophil ratio
RF: Random Forest
LR: Logistic regression
SVM: Support vector machine
GBM: Gradient boosting machine
NN: Neural network
XGBoost: Extreme gradient boosting
KNN: K-nearest neighbors
